# Diagnosing bovine parafilariosis: utility of the cytochrome *c* oxidase subunit 1 gene and internal transcribed spacer region for PCR detection of *Parafilaria bovicola* in skin biopsies and serohemorrhagic exudates of cattle

**DOI:** 10.1186/s13071-019-3838-4

**Published:** 2019-12-11

**Authors:** Andreas W. Oehm, Alexander Stoll, Cornelia Silaghi, Annette Pfitzner-Friedrich, Gabriela Knubben-Schweizer, Christina Strube

**Affiliations:** 1 Clinic for Ruminants with Ambulatory and Herd Health Services, Ludwig-Maximilians-Universität Munich, Sonnenstrasse 16, 85764 Oberschleissheim, Germany; 2 Institute for Comparative Tropical Medicine and Parasitology, Ludwig-Maximilians-Universität Munich, Leopoldstrasse 5, 80802 München, Germany; 3 Institute of Infectology (IMED), Friedrich-Loeffler-Institute, Südufer 10, 17493 Greifswald-Insel Riems, Germany; 4 Institute for Parasitology, Centre for Infection Medicine, University of Veterinary Medicine Hannover, Buenteweg 17, 30559 Hanover, Germany

**Keywords:** *Parafilaria bovicola*, Cattle, Filarial nematode, Filarioidea, Microfilariae, PCR, *cox*1, ITS

## Abstract

**Background:**

*Parafilaria bovicola* (Nematoda: Filariidae) causes cutaneous bleedings in bovine species. Flies serve as intermediate hosts. In recent years, reports on bovine parafilariosis have become more frequent, corroborating the necessity of reliable diagnostic interventions especially since no molecular or serological test has been available. We aimed to establish a polymerase chain reaction assay to detect DNA of *P. bovicola* in flies, skin biopsies and serohemorraghic exudates of bleeding spots.

**Methods:**

PCRs targeting the cytochrome *c* oxidase subunit 1 (*cox*1) gene and the internal transcribed spacer region (ITS) of the ribosomal RNA gene cluster were evaluated for their diagnostic sensitivity as well as performance and specificity on biopsy and serohemorrhagic exudate samples from *P. bovicola-*infected cattle.

**Results:**

Using serohemorrhagic exudates (*n* = 6), biopsies (*n* = 2) and flies (*n* = 1), the PCR targeting the *cox*1 gene resulted in a gel band of almost 700 bp. Cloning, sequencing, and removal of primer sequences yielded a 649-bp fragment of the *P. bovicola cox*1 gene. The PCR targeting the ITS region showed a band of about 1100 bp. Cloning, sequencing, and removal of primer sequences resulted in a 1083 bp stretch of the *P. bovicola* ITS region. Testing samples from presumably affected animals, the *cox*1-PCR resulted in bands with the expected size and they were all confirmed as *P. bovicola* by sequencing. In contrast, the ITS-PCR proved to be less sensitive and less specific and additionally amplified the ITS region of *Musca domestica* or buttercup DNA. When analysing for sensitivity, the *cox*1-PCR yielded visible bands up to 2 ng of genomic DNA, whereas the ITS-PCR produced bands up to 3 ng. In a plasmid dilution series, the minimum number of target DNA copies was 10^2^ for the *cox*1-PCR and 10^1^ in the ITS-PCR.

**Conclusions:**

The evaluated *cox*1-PCR enables reliable detection of *P. bovicola* DNA in skin biopsies and serohemorrhagic exudates. This PCR and, to a limited extent, the ITS-PCR, may help evaluate different therapeutic approaches. Furthermore, the *cox*1-PCR may be useful for epidemiological studies on the geographical distribution of *P. bovicola*. Further understanding of the epidemiology of this parasite will help develop and implement effective control strategies.
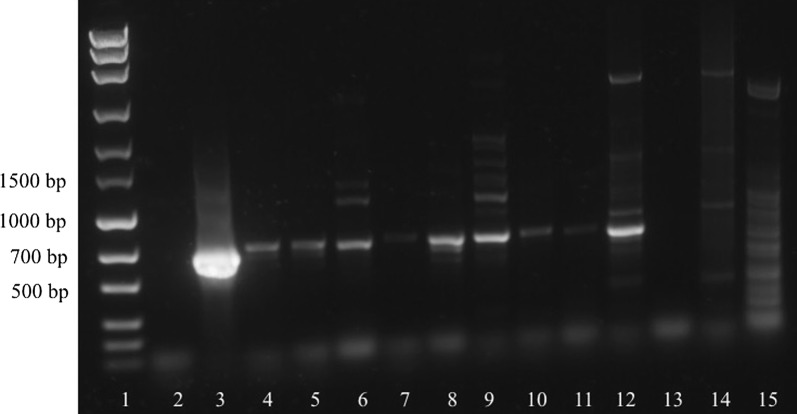

## Background

*Parafilaria bovicola* is a filarial nematode that causes signs of “cutaneous bleeding” in affected bovine species. In 1934, Tubangui [1] and de Jesus [2] were the first to give a profound parasitological description of this parasite. While the localization of the males is still mainly unknown, adult ovoviviparous females of *P. bovicola* live encapsulated in cutaneous and subcutaneous nodules [3] which they penetrate temporarily to oviposit through a fistulous tract to the cutaneous surface of their host. These lesions release serohemorrhagic exudates containing a mixture of eggs and microfilariae (first larval stage, L1). Microfilariae are ingested by intermediate hosts, such as *Musca autumnalis* in Europe, nourishing on the exudates [4]. In the intermediate host, ingested L1 develop into infective larvae (L3), for different periods (2–3 weeks) depending on environmental temperature [3]. L3 exit through the proboscis of the flies while these feed on secretions of mucous membranes of cattle and penetrate these. Subsequently, the migration of L3 larvae through subcutaneous tissues, development to adult stages and appearance of first bleeding spots require 7–9 months [3, 5, 6].

Infection with *P. bovicola* is characterized by a seasonal occurrence of intermittent skin bleedings especially in the collar, scapular, withers and thoracic region [3, 4, 6–9] and causes severe eosinophilic inflammation of the skin [2, 3, 10, 11], which may affect adjacent muscle tissues [7, 12]. Myiasis, expanded cutaneous ulcerations or necrosis, respectively, and secondary abscesses have equally been reported [2, 13, 14]. *Parafilaria*-induced lesions have even been detected in sub-pleural, abdominal, mediastinal and perirenal tissues [15]. Lesions of this kind do often lead to condemnation of the entire carcass affected. Infested cattle show typical signs of infirmity [2].

Considerable economic losses have been demonstrated in meat production due to increased carcass trimming and reduced leather quality [12, 16–18]. Further studies have reported a marked decrease in milk yield and weight loss as a consequence of discomfort in infected cows as well [8, 13, 18, 19].

During the last few years there has been a remarkable increase of cases of parafilariosis in cattle with reports showing the presence of *P. bovicola* in Austria [20], Belgium [10], Germany [11], Italy [3] and The Netherlands [4]. Direct observation of bleeding spots and/or the presence of adult worms in carcasses or biopsies have so far been used to diagnose parafilariosis in cattle. Furthermore, microfilariae or larvated eggs can be detected in the serohemorrhagic exudate using microscopy.

However, the current detection methods for parafilariosis in cattle entail the risk of false diagnoses and thus are not satisfactory. So far, no molecular or serological test has been available to confirm diagnosis. Therefore, the aim of the present study was to evaluate a polymerase chain reaction assay (PCR) for detection of *P. bovicola* DNA in skin biopsies and serohemorrhagic exudates of bleeding spots to allow for fast and reliable diagnosis of clinical cases.

## Methods

### Collection of *P. bovicola* adults

Two cows displaying conspicuous bleeding spots were selected for the collection of skin biopsies or *P.* *bovicola* adults, respectively. The sites were clipped, cleaned using iodine soap and disinfected with 70% ethanol. A volume of 15.0 ml of a local anaesthetic (lidocainhydrochloride) was injected subcutaneously around the site and after 10 min 70% ethanol was applied again.

An almost 2.0 cm long, white worm was observed in the center of the swollen skin site, trying to leave the skin. The worm was gently removed manually and transferred to 70% ethanol in a 10 ml Falcon tube.

Another worm was collected as described above during preparation for a biopsy of a bleeding spot in a dairy cow. *Parafilaria bovicola* abruptly pervaded the skin when the site was manipulated and remained sticking on the skin surface. The worm was collected manually and stored in 70% ethanol at 4 °C. Both nematodes were examined morphologically and identified as female specimens of *P. bovicola* [21].

### Collection of serohemorrhagic exudates, skin biopsies, blood and flies

Fresh (hereafter referred to as “liquid exudate”) or dry samples (hereafter referred to as “dried exudate”) of the bleeding spots of presumably affected animals as well as of those animals, from which the two adult specimens of *P. bovicola* were isolated, were collected. These samples were transferred to 70% ethanol and kept at − 20 °C.

To obtain biopsies, conspicuous skin sites were prepared as described above and an individually wrapped, disposable and sterile biopsy punch of 8 mm in diameter (Jørgen Kruuse A/S, Langeskov, Denmark) was used to cut out a cylindrical piece of skin. Biopsies were conserved in 70% ethanol and frozen at − 20 °C.

As negative controls, EDTA blood and skin biopsies from clinically sound cows at the Clinic for Ruminants and at the Livestock Center of the Ludwig-Maximilians-Universität, Munich, Germany, were collected. This was in compliance with animal welfare standards. Additionally, flies (*Musca* sp.) were caught at presumably affected farms as potential sources of contaminating DNA in bleeding spots as well as potential *P. bovicola* intermediate host.

### DNA extraction and PCR of adult *P. bovicola*

Genomic DNA was isolated from an approximately 10 mm piece of the adult worm using the NucleoSpin^®^ Tissue Kit (Macherey-Nagel, Düren, Germany). Subsequent PCRs targeted the cytochrome *c* oxidase subunit 1 (*cox*1) gene by using the primers COIintf and COIintR [22] as well as the internal transcribed spacer region (ITS1 region, *5.8S* rDNA and ITS2 region with flanking *18S* and *28S* rDNA sequences) using the primers NC5 and NC2 [23]. The reaction set-up for both PCRs comprised 25 μl, containing 0.5 µl DreamTaq DNA Polymerase (5 U/μl) (Thermo Fisher Scientific, Schwerte, Germany), 2.5 µl 10× DreamTaq buffer, 0,5 μl dNTP mix (10 mM each), 1 μl of each forward and reverse primer (10 µM each) and 1 μl DNA template. Thermocycling conditions targeting the *cox*1 gene were as follows: initial denaturation at 95 °C for 3 min, 30 cycles of 95 °C for 30 s, 55 °C for 30 s, 72 °C for 30 s and a final elongation step at 72 °C for 10 min. Thermocycling conditions targeting the ITS-region were: initial denaturation at 95 °C for 3 min, 30 cycles of 95 °C for 45 s, 50 °C for 45 s, 72 °C for 90 s and a final elongation step at 72 °C for 10 min. The PCR products were visualized on a 1% agarose gel.

Afterwards, the amplicons were inserted into the pCR4™4-TOPO^®^ vector and cloned into One Shot^®^ TOP10 chemically competent *E. coli* using the TOPO^®^ TA Cloning^®^ Kit for Sequencing (Invitrogen, Schwerte, Germany). Plasmid DNA was obtained using the NucleoSpin^®^ Plasmid Kit (Macherey-Nagel) following the manufacturer’s recommendations. Subsequently, the inserts were custom-sequenced (Seqlab Sequence Laboratories, Göttingen, Germany) and analysed by BLASTn against publicly available nucleotide sequences. After removal of primer sequences, the newly generated sequences were deposited in the GenBank database under the accession numbers MG983750 and MG983751.

### Sensitivity of the *cox*1- and ITS-PCR

To test the analytic sensitivity of the *cox*1- and ITS-PCR, dilution series of adult worm genomic DNA and plasmids inserting the *cox*1- and ITS amplification products (see above) were generated. To prepare dilutions, the yield of isolated genomic DNA and plasmid DNA was determined by measuring the absorbance at 260 nm with the NanoDrop™ 1000 spectrophotometer (PEQLAB Biotechnologie GmbH, Erlangen, Germany). For testing genomic DNA dilutions as template, PCRs were performed as described above using the following amounts of DNA template: 100 ng, 50 ng, 20 ng, 10 ng to 1 ng (1000 pg), 100 pg, 50 pg, 10 pg to 1 pg. For testing plasmid DNA, 10-fold serial dilutions ranging from 10^9^ to 10^0^ target copies per PCR reaction were prepared. Each 10 µl amplification product was run on a 1% agarose gel stained with GelRed^®^ (Biotium Inc., Fremont, CA, USA) and visualized under UV light.

### DNA extraction and PCR of serohemorrhagic exudates, skin biopsies, blood and flies

For an initial screening whether the *cox*1- and ITS-PCRs are suitable to detect *P. bovicola* DNA in samples of presumably affected bovines, genomic DNA was isolated with the NucleoSpin^®^ Tissue Kit from 100 µl and 200 µl native liquid exudate of two cows each. Additionally, liquid exudate fixed in 70% ethanol from one of the cows was subjected to DNA isolation. PCRs targeting the *cox*1 gene and ITS region were conducted as described above, with the exception that 2 µl genomic DNA was used as template. Genomic DNA from the adult *P. bovicola* worm was used as a positive control. From each PCR product, 10 µl were loaded on a 1% agarose gel to visualize the amplicons.

For final experiments, genomic DNA was isolated from 100 µl liquid exudate (native or fixed with ethanol), 100 µg dried exudate, 20–30 µg skin biopsy and one *Musca* sp. fly caught at an affected farm. Again, 2 µl were used as PCR template and 10 µl PCR product was loaded on a 1% agarose gel to visualize the amplicons. Selected (ITS-PCR) or all (*cox*1-PCR) bands at approximately the right size were excised and custom-sequenced (Seqlab Sequence Laboratories, Göttingen, Germany). Obtained sequences were compared with the sequences generated from the adult *P. bovicola* specimen and by BLASTn against public databases.

## Results

### Amplification of adult *P. bovicola* DNA

The PCR targeting the *cox*1 gene resulted in a gel band of almost 700 bp in size. Cloning and sequencing revealed an amplification product of 689 bp and after removal of the primers, sequences a 649-bp fragment of the *cox*1 gene of *P. bovicola* was obtained. BLASTn sequence comparison resulted in the *cox*1 gene of *Onchocerca gibsoni* as a top hit (GenBank: AJ271616; identity: 88%; query cover, QC: 98%; e-value: 0.0), followed by *Spirocerca lupi* (GenBank: KC305876; identity: 87%; QC: 100%; e-value: 0.0) and *Dirofilaria repens* (GenBank: KR998259; identity: 87%; QC: 99%; e-value: 0.0).

The ITS-PCR showed a band of about 1100 bp in size. Cloning and sequencing resulted in an amplification product of 1129 bp and after removal of the primer sequences in a 1083-bp fragment. The closest match *via* BLASTn search was the ITS region of *Onchocerca fasciata* (GenBank: JQ316671, identity: 77%; QC: 42%; e-value: 1e−80), followed by *Brugia pahangani* (GenBank: EU373654; identity: 75%; QC: 43%; e-value: 3e−75) and *Parabronema skrjabini* (GenBank: EU375510; identity: 76%; QC: 41%; e-value: 3e−75).

### Sensitivity of the *cox*1- and ITS-PCR

**Fig. 1:**
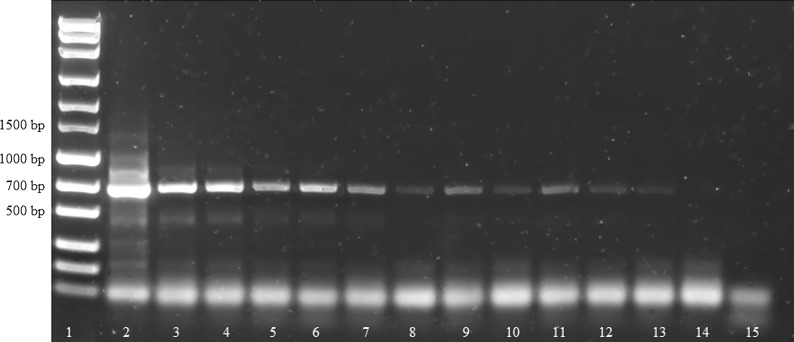
Sensitivity of the *cox*1-PCR using genomic DNA dilutions as template. Lane 1: marker (MassRuler^®^ Express Forward DNA Ladder Mix, Thermo Fisher Scientific); Lane 2: 1000 pg genomic DNA; Lane 3: 100 pg genomic DNA; Lane 4: 50 pg genomic DNA; Lane 5: 10 pg genomic DNA; Lane 6: 9 pg genomic DNA; Lane 7: 8 pg genomic DNA; Lane 8: 7 pg genomic DNA; Lane 9: 6 pg genomic DNA; Lane 10: 5 pg genomic DNA; Lane 11: 4 pg genomic DNA; Lane 12: 3 pg genomic DNA; Lane 13: 2 pg genomic DNA; Lane 14: 1 pg genomic DNA; Lane 15: no-template control

**Fig. 2:**
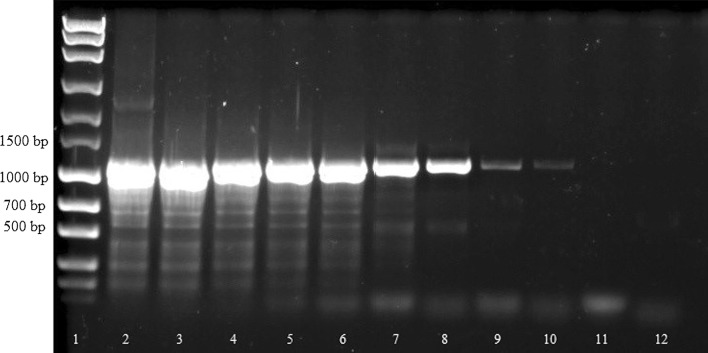
Sensitivity of the ITS-PCR using serial plasmid dilutions as template. Lane 1: marker (MassRuler^®^ Express Forward DNA Ladder Mix, Thermo Fisher Scientific); Lane 2: 10^9^ target copies; Lane 3: 10^8^ target copies; Lane 4: 10^7^ target copies; Lane 5: 10^6^ target copies; Lane 6: 10^5^ target copies; Lane 7: 10^4^ target copies; Lane 8: 10^3^ target copies; Lane 9: 10^2^ target copies; Lane 10: 10^1^ target copies; Lane 11: 10^0^ target copies; Lane 12: no-template control

Testing the analytic sensitivity of the PCRs revealed that the *cox*1-PCR produced visible bands with as little as 2 ng of genomic DNA template (Fig. 1), while the ITS-PCR produced bands with as little as 3 ng of genomic DNA template. When using plasmid dilution series to determine the required minimum number of target DNA copies, *cox*1-PCR resulted in bands with a minimum of 10^2^ target copies, while the minimum copy number was 10^1^ in the ITS-PCR (Fig. 2).

### PCR of serohemorrhagic exudates, skin biopsies, blood and flies

**Fig. 3:**
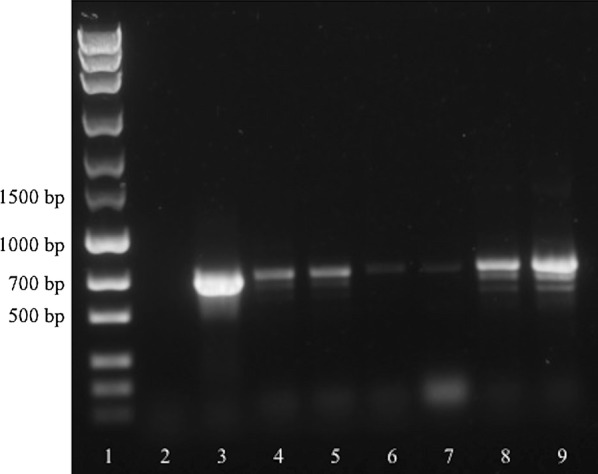
Initial screening amplification of liquid exudate samples using *cox*1-PCR. Lane 1: marker (MassRuler™ Express Forward DNA Ladder Mix, Thermo Fisher Scientific); Lane 2: no-template control; Lane 3: positive control; Lane 4: 100 µl native liquid exudate (cow 1); Lane 5: 200 µl native liquid exudate (cow 1); Lane 6: 100 µl liquid exudate fixed in 70% ethanol (cow 2); Lane 7: 200 µl liquid exudate fixed in 70% ethanol (cow 2); Lane 8: 100 µl native liquid exudate (cow 2); Lane 9: 200 µl native liquid exudate (cow 2)

**Fig. 4:**
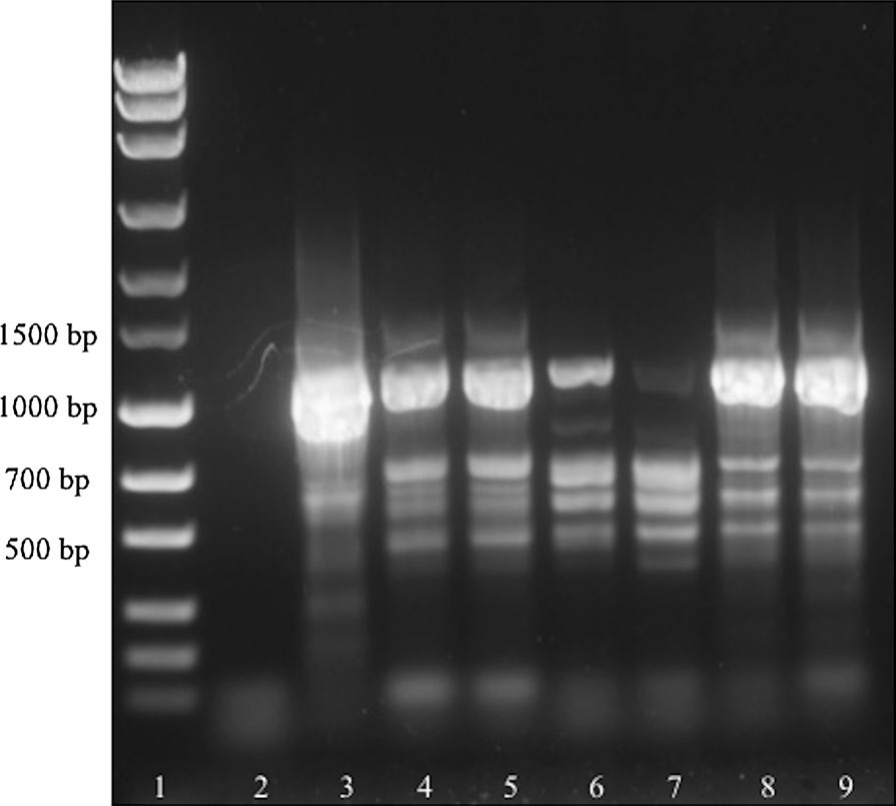
Initial screening amplification of liquid exudate samples using ITS-PCR. Lane 1: marker (MassRuler^®^ Express Forward DNA Ladder Mix, Thermo Fisher Scientific); Lane 2: no-template control; Lane 3: positive control; Lane 4: 100 µl native liquid exudate (cow 1); Lane 5: 200 µl native liquid exudate (cow 1); Lane 6: 100 µl liquid exudate fixed in 70% ethanol (cow 2); Lane 7: 200 µl liquid exudate fixed in 70% ethanol (cow 2); Lane 8: 100 µl native liquid exudate (cow 2); Lane 9: 200 µl native liquid exudate (cow 2)

A total of six samples of serohemorrhagic exudate, two biopsies and one fly were included in this analysis. Initial screening *cox*1- and ITS-PCRs using different amounts of native and fixed liquid exudate for DNA isolation revealed bands at the expected size of about 700 bp (*cox*1-PCR, Fig. 3) and 1100 bp (ITS-PCR, Fig. 4), respectively, for all PCR reactions. When comparing band intensity, the amount of 100 µl liquid exudate used for DNA isolation was not inferior to 200 µl liquid exudate. However, the bands originating from liquid exudate samples fixed (and thus diluted) in 70% ethanol were rather faint compared with the native exudate samples.

**Fig. 5:**
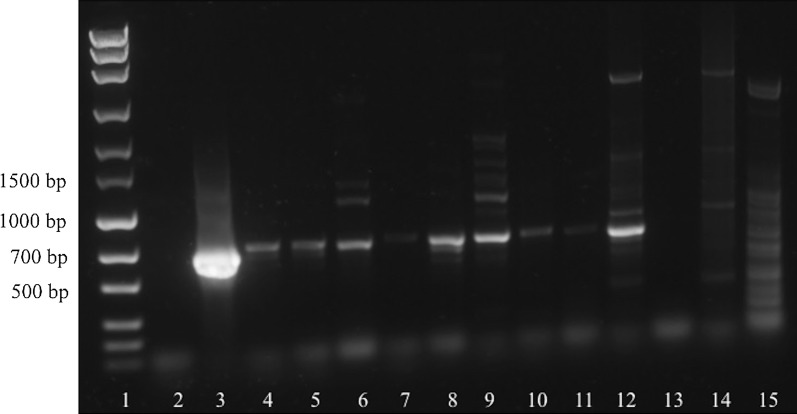
Detection of *P. bovicola* in samples from presumably affected (Lanes 4–12) and control animals (Lanes 13–14) and a *Musca* sp. fly using *cox*1-PCR. Lane 1: marker (MassRuler^®^ Express Forward DNA Ladder Mix, Thermo Fisher Scientific); Lane 2: no-template control; Lane 3: positive control; Lane 4: native liquid exudate (cow 1); Lane 5: native liquid exudate (cow 3); Lane 6: native liquid exudate (cow 4); Lane 7: liquid exudate fixed in 70% ethanol (cow 2); Lane 8: native liquid exudate (cow 2); Lane 9: native liquid exudate (cow 5); Lane 10: dry exudate (cow 2); Lane 11: dry exudate (cow 6); Lane 12: skin biopsy fixed in 70% ethanol (cow 7); Lane 13: EDTA blood (control cow 1); Lane 14: skin biopsy (control cow 2); Lane 15: *Musca* fly

**Fig. 6:**
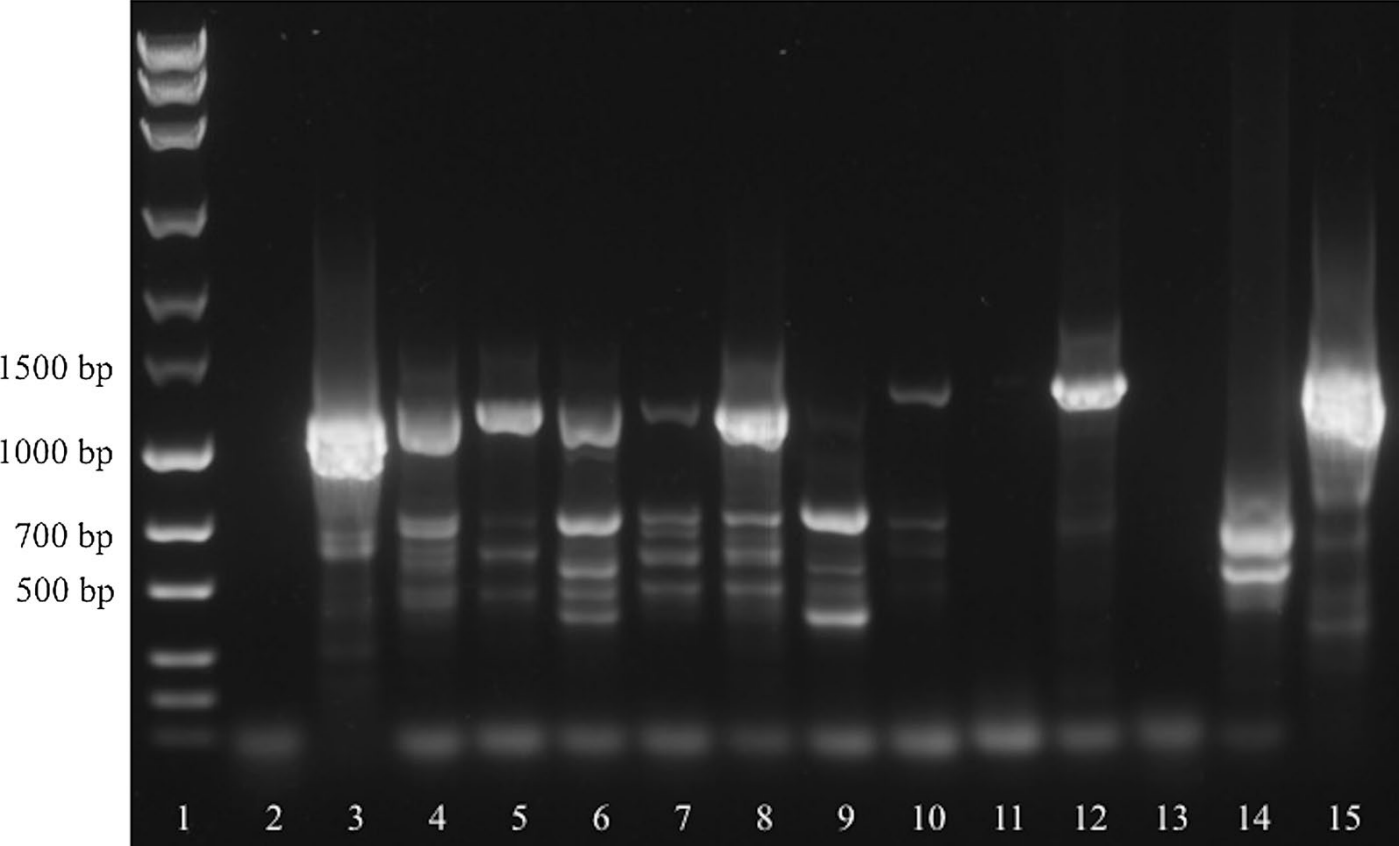
Detection of *P. bovicola* in samples from presumably affected (Lanes 4–12) and control animals (Lanes 13–14) and a *Musca* sp. fly using ITS-PCR. Lane 1: marker (MassRuler^®^ Express Forward DNA Ladder Mix, Thermo Fisher Scientific); Lane 2: no-template control; Lane 3: positive control; Lane 4: native liquid exudate (cow 1); Lane 5: native liquid exudate (cow 3); Lane 6: native liquid exudate (cow 4); Lane 7: liquid exudate fixed in 70% ethanol (cow 2); Lane 8: native liquid exudate (cow 2); Lane 9: native liquid exudate (cow 5); Lane 10: dry exudate (cow 2); Lane 11: dry exudate (cow 6); Lane 12: skin biopsy fixed in 70% ethanol (cow 7); Lane 13: EDTA blood (control cow 1); Lane 14: skin biopsy (control cow 2); Lane 15: *Musca* sp. fly

When testing all available samples from presumably affected animals, *cox*1*-*PCR resulted in a band at the expected size for each sample. Samples from control animals as well as the *Musca* sp. fly from a presumably affected farm remained negative (Fig. 5). In contrast, ITS-PCR did not reveal a band at the expected size or no bands at all in two samples (Lanes 9, 11, Fig. 6). Again, samples from control animals remained negative, but amplification of the *Musca* sp. fly DNA resulted in a strong band at the expected size (Fig. 6).

### Sequencing of amplification products from serohemorrhagic exudates, skin biopsies and flies

Sanger sequencing was performed with all *cox*1-PCR amplification products (or excised gel bands, respectively) of the presumably affected animals (Lanes 4–12 in Fig. 5) and with the prominent bands at *c.*1100 bp from Lanes 4, 12 and 14 (*Musca* sp. fly) as well as the prominent short band at *c.*700 bp (Lane 9 in Fig. 6) from the ITS-PCR products. In sequences alignment search, all obtained sequences perfectly matched the *P. bovicola cox*1 sequence generated from the adult worm (see above, GenBank: MG983751) and the bands at the expected size of the presumably affected cows (Lanes 4, 12) matched the *P. bovicola* ITS sequence generated from the adult worm (see above, GenBank: MG983750). However, the band of the *Musca* sp. fly (Lane 15) matched a *Musca domestica* ITS sequence (GenBank: Z28417) and the short 700 bp band (Lane 9) of a presumably affected cow was identified as ITS region of a buttercup species (*Ranunculus subcorymbosus*, GenBank: FM242810).

## Discussion

Cutaneous bleeding in cattle always represents an exceptional situation in daily veterinary practice work. In recent years, reports on the occurrence of cases of bleeding cattle have become more abundant [3, 4, 24]. It is unclear whether this refers to an enhanced interest in bleeding cattle after the emergence of bovine neonatal pancytopenia in 2006 and 2007 [25–27] or to an actual increase in the prevalence of *P. bovicola* in cattle. Regarding the latter, Brooks et al. [28] have elucidated that the emergence of “new diseases” can be traced back to either an introduction into previously non-endemic areas or to an environmental change in regions they spread to. Climate change has been demonstrated to have pronounce effects on the epidemiology, spatial spread and seasonal dynamics of parasite populations [29, 30]. Parasite stages that are dependent on intermediate hosts are distinctively sensitive to climatic alterations influencing themselves as well as the abundance, resilience and reproduction of their intermediate hosts [31]. Therefore, reduced temperature limitations may enable new pathogen species to establish in hitherto free areas [28, 31–33].

To date, diagnosis of bovine parafilariosis has been restricted to the presence and interpretation of clinical signs, which, however, are not a confirmation or detection of adult worms, eggs or microfilariae, from freshly active bleeding spots shortly after start of the bleeding [34]. Isolation of adult specimens or searching for eggs and microfilariae in serohemorrhagic exudates is fairly unreliable [9, 19, 35]. According to our own experience, adult *P. bovicola* are very elusive and hence their collection is challenging and is successful only in very few cases. This is further corroborated by Borgsteede et al. [4], who reported that no eggs or microfilariae were detected in the exudate of a breeding bull, even though female worms containing large numbers of eggs with microfilariae were detected during necropsy. Sundquist et al. [36, 37] developed an ELISA based on native exoantigen of the parasite, which allowed for sensitive and specific detection of antibodies against *P. bovicola* but required a continuous supply and thus continuous availability of worms for coating of the ELISA plates. This proved to be impractical and the method could not be widely established. Nevertheless, serological assays detecting antibodies against exoantigens of adult worms may have the limitation of a lag-phase, in which egg-laying adult worms may be present but antibodies have not yet been developed.

Thus, the aim of the present study was to evaluate a PCR assay for detection of *P. bovicola* DNA in samples of infected cattle to provide an easy and reliable diagnostic approach for bovine parafilariosis. Casiraghi et al. [22] have shown the phylogenetic relationships between filarial nematodes based on mitochondrial DNA, i.e. *cox*1 gene sequences. Mitochondrial DNA is phylogenetically conserved within specimens of a taxon and the *cox*1 gene has thus been used in a range of taxonomic studies to disentangle phylogenetic relationships among species [38–42]. Besides mitochondrial DNA, the ribosomal ITS region is an excellent target to discriminate nematodes (reviewed in Blouin [43]) and has been frequently used for filarial species delineation and identification [4, 43–46]. Therefore, the *cox*1 gene as well as the ITS region were chosen as targets in our PCR approach and compared regarding their suitability as diagnostic tools for bovine parafilarosis. Both *cox*1- and ITS-PCR amplified genomic DNA isolated from an adult worm, enabled us to provide the first *P. bovicola* sequences in public databases. Since *P. bovicola* is classified within the superfamily Filarioidea, it was at first glance not surprising that for both, the *cox*1 and ITS sequences, an *Onchocerca* species was the top hit in sequence identity search. However, the genus *Onchocerca* belongs to the family Onchocercidae, while the genus *Parafilaria* is a member of the family Filariidae. For the Filariidae, a few *cox*1 sequences are publicly available for species of the genus *Filaria*. Interestingly, respective top matches from this family (*Filaria* sp., KJ612514; and *Filaria martis*, KU761590) showed lower sequence identity (KJ612514: identity: 85%; QC: 97%; e-value: 0.0; KU761590: identity: 82%; QC: 99%; e-value: 0.0) with the *cox*1 sequence for *P. bovicola* than various members of the family Onchocercidae or even e.g. *S. lupi* of the superfamily Spiruroidea. Overall, no member of the family Filariidae was amongst the available top 100 BLAST hit descriptions.

Testing the analytical sensitivity of the *cox*1- and ITS-PCR showed that both PCRs are very sensitive using plasmids inserting the target sequence as a simple template. Here, the PCRs produced visible bands with 100 and 10 target copies, respectively. Similarly, when using genomic *P. bovicola* DNA as a more complex template, both PCRs performed well with a detection limit of 2–3 pg template DNA. Such successful amplification allowed us to transfer the PCR protocols to diagnostic material from presumably *P. bovicola* infected cattle, a quite complex template, because parasite DNA is mixed (to a more or less large degree) with host DNA. As diagnostic material collection from cutaneous bleedings is limited in its amount, we compared PCR efficiency after DNA extraction from 100 µl *vs* 200 µl liquid exudate. Interestingly, gel band intensity was mostly unaffected by the amount used for DNA extraction, but fixation of the exudate in 70% ethanol significantly reduced PCR efficiency. Since the reduction in band intensity was disproportionately high, the reason is most likely less efficient DNA isolation due to ethanol precipitation of DNA rather than a dilution effect. Based on these findings, a sample volume of 100 µl serohemorrhagic exudate is sufficient for PCR diagnostics, but samples should not be conserved with ethanol but rather kept cool or frozen until analysis.

When analyzing all available samples of presumably *P. bovicola*-affected cows, *cox*1-PCR reliably detected all of them as positive. Again, the ethanol-fixed liquid exudate sample resulted in a faint band only, whereas the utilized 20 µg ethanol-fixed skin biopsy showed a prominent signal, indicating that this biopsy contained more eggs or microfilariae than the exudate, which originated from a different cow. Noteworthy, amplification efficacy and thus band intensity of dried exudate was inferior to liquid exudate. Consequently, liquid serohemorrhagic exudate or skin biopsies should be collected for PCR diagnosis whenever possible.

Results of the ITS-PCR were inferior compared to *cox*1-PCR, as no amplification signal was observed with one of the two dried exudate samples and, furthermore, one liquid exudate sample resulted in a prominent band below the expected amplicon size, whereas the band at the correct size could only be suspected (cf. Lane 9, Fig. 6). Sequencing showed that the prominent band represented the ITS sequence of a buttercup. This can be explained by adhering or trapped buttercup seeds or small leaf particles in the liquid exudate when cows lay down on the pasture to ruminate. Moreover, ITS-PCR resulted in a very strong band at the expected *P. bovicola* amplicon size when amplifying DNA isolated from a *Musca* sp. fly from an affected farm. Sequencing revealed this amplification product as an ITS sequence of the housefly *Musca domestica*. This result is a major shortcoming of the ITS-PCR, as flies may deposit their eggs in the wounds or exudate, leading to false positive results in *P. bovicola* diagnosis by ITS-PCR. Furthermore, this undesired amplicon of *Musca* sp. DNA at the expected *P. bovicola* amplicon size, excludes the ITS-PCR from epidemiological studies on the prevalence of infected flies in affected farms.

From a therapeutic point of view, our *cox*1-PCR and, to a limited extent, the ITS-PCR, may serve as a potential control for the effects of several therapeutic approaches for parafilariosis in cattle. In this context, Torgerson et al. [17] reported that the use of anthelmintic drugs is not sufficiently efficient against *P. bovicola.* The *cox*1-PCR method described in this paper will facilitate the evaluation of the outcomes of implementing different anthelmintic compounds in infected bovines and their effects on different developmental stages of *P. bovicola* and therefore help diagnosing and monitoring with this emerging parasite. Furthermore, the presented PCR assay provides a non-invasive tool to further investigate the biology and presence of this emerging parasite, since it has not been yet understood how long and to what extent are larval stages persistent in flies. This is of crucial importance since intermediate hosts are a central element in the epidemiology of *P. bovicola* and Nevill et al. [44] pointed out that face flies of the genus *Musca* are extremely competent in transmission dynamics. Van Dijk et al. [29] illustrated that helminths are able to rapidly adapt to new environmental conditions, which can be attributed to their relatively short generation times. As a consequence of milder climatic conditions, helminth evolution may be accelerated to an extent mammal host are not able to keep up with. This puts further emphasis on the necessity of improved diagnostic approaches for surveillance of parasite population dynamics, evaluation of parasite burdens in livestock and therapeutic efficiency of anthelmintics [29]. Finally, the evaluated *cox*1-PCR represents a tool for epidemiological studies to monitor the geographical expansion of *P. bovicola* in previously non-endemic regions by using bovine samples or intermediate host flies. Further understanding of the epidemiology of this emerging parasite will help develop and implement effective control strategies to minimise the impact on productivity and welfare of cattle.

## Conclusions

The *cox*1-PCR presented here enables reliable detection of *P. bovicola* DNA in presumably affected animals. From a therapeutic point of view, *cox*1-PCR and, to a limited extent, the ITS-PCR, may serve as potential controls for the effects of several therapeutic approaches for parafilariosis in cattle. Finally, the evaluated *cox*1-PCR represents a tool for epidemiological studies on the geographical distribution of *P. bovicola* by using bovine samples or intermediate host flies. Further understanding of the epidemiology of this emerging parasite will help develop and implement effective control strategies to minimise the impact on productivity and welfare of cattle.

## Data Availability

Data supporting the conclusions of this article are included within the article. The newly generated sequences were deposited in the GenBank database under the accession numbers MG983750 and MG983751. The datasets used and/or analysed during the present study are available from the corresponding author upon reasonable request.

## Abbreviations

BLAST: Basic Local Alignment Search Tool
bp: base pair(s)
*cox*1: cytochrome *c* oxidase subunit 1 gene
DNA: deoxyribonucleic acid
ITS: internal transcribed spacer region
PBS: phosphate-buffered saline
PCR: polymerase chain reaction
